# Clinical, histopathological and genetic characterisation of oculoskeletal dysplasia in the Northern Inuit Dog

**DOI:** 10.1371/journal.pone.0220761

**Published:** 2019-08-15

**Authors:** Renata Stavinohova, Claudia Hartley, Louise M. Burmeister, Sally L. Ricketts, Louise Pettitt, Roser Tetas Pont, Rebekkah J. Hitti, Ellen Schofield, James A. C. Oliver, Cathryn S. Mellersh

**Affiliations:** 1 Unit of Comparative Ophthalmology, Centre for Small Animal Studies, Animal Health Trust, Kentford, Newmarket, Suffolk, United Kingdom; 2 Kennel Club Genetics Centre, Animal Health Trust, Kentford, Newmarket, Suffolk, United Kingdom; Justus Liebig Universitat Giessen, GERMANY

## Abstract

Seven Northern Inuit Dogs (NID) were diagnosed by pedigree analysis with an autosomal recessive inherited oculoskeletal dysplasia (OSD). Short-limbed dwarfism, angular limb deformities and a variable combination of macroglobus, cataracts, lens coloboma, microphakia and vitreopathy were present in all seven dogs, while retinal detachment was diagnosed in five dogs. Autosomal recessive OSD caused by *COL9A3* and *COL9A2* mutations have previously been identified in the Labrador Retriever (dwarfism with retinal dysplasia 1—*drd1*) and Samoyed dog (dwarfism with retinal dysplasia 2—*drd2*) respectively; both of those mutations were excluded in all affected NID. Nine candidate genes were screened in whole genome sequence data; only one variant was identified that was homozygous in two affected NID but absent in controls. This variant was a nonsense single nucleotide polymorphism in *COL9A3* predicted to result in a premature termination codon and a truncated protein product. This variant was genotyped in a total of 1,232 dogs. All seven affected NID were homozygous for the variant allele (*T/T*), while 31/116 OSD-unaffected NID were heterozygous for the variant (*C/T*) and 85/116 were homozygous for the wildtype allele (*C/C*); indicating a significant association with OSD (*p* = 1.41x10^-11^). A subset of 56 NID unrelated at the parent level were analysed to determine an allele frequency of 0.08, estimating carrier and affected rates to be 15% and 0.6% respectively in NID. All 1,109 non-NID were *C/C*, suggesting the variant is rare or absent in other breeds. Expression of retinal mRNA was similar between an OSD-affected NID and OSD-unaffected non-NID. In conclusion, a nonsense variant in *COL9A3* is strongly associated with OSD in NID, and appears to be widespread in this breed.

## Introduction

Oculoskeletal dysplasia (OSD) is an inherited disease characterised by skeletal and ocular defects and has previously been described in the Labrador Retriever and Samoyed dog breeds [1–6]. Affected dogs have skeletal abnormalities, characterised by disproportionate short-limbed dwarfism (short appendages with a normal axial skeleton) and skeletal dysplasia, characterised by retarded bone growth, deformative bone growth, angular limb deformities, bone dysplasia and degenerative arthropathy [1–7]. Ocular defects consist of combinations of corneal opacities, axial myopia/macroglobus, varying degrees of cataract formation, persistent hyaloid artery remnants, vitreous abnormalities, alteration in optic nerve colour and size, tapetal hypoplasia, retinal dysplasia, retinal tears and detachment [1–7]. Variants associated with OSD have been identified in *COL9A3* in the Labrador Retriever and in *COL9A2* in the Samoyed dog, termed *drd1* and *drd2* (dwarfism with retinal dysplasia 1 and 2) respectively [5]. The mode of inheritance of the complete phenotype is reported to be autosomal recessive in both breeds [5]. However, it is also reported that the ocular lesions may represent a dominant trait with incomplete penetrance in the Labrador Retriever; skeletally normal heterozygotes can have either no ophthalmic abnormalities or vitreal membranes, vitreous degeneration, focal/multifocal retinal folds or geographic retinal dysplasia [2, 5]. The main ocular histopathological findings in the Labrador Retriever homozygous for the *COL9A3* mutation are marked vitreous abnormalities, vitreal traction creating retinal tears, liquefied vitreous, cellular vitreoretinal proliferation associated with retinal tears and detachment, and axial myopia [1, 2, 4, 8].

The clinical and pathological phenotypes of OSD in the Labrador Retriever and Samoyed dog resemble a number of human hereditary arthro-ophthalmopathies, including Stickler syndrome, Kniest dysplasia and Marshall syndrome [9–25]. The six types of Stickler syndrome (STL type I-VI) present with a variety of clinical signs and symptoms, which vary widely among affected individuals. Stickler syndrome types I, II and III are inherited in an autosomal dominant fashion and have been associated with mutations in *COL2A1*, *COL11A1* and *COL11A2* [9–11], while types IV, V and VI are inherited in an autosomal recessive fashion and have been associated with mutations in *COL9A1*, *COL9A2* and *COL9A3* [12–14, 17]. Clinical signs of STL types I, II, IV and V include myopia, cataracts, vitreoretinal degeneration, posterior chorioretinal atrophy, rhegmatogenous retinal detachment, midface hypoplasia with cleft palate, hearing loss and skeletal dysplasia [12, 13, 15–20].

Kniest dysplasia is caused by autosomal dominant mutations in *COL2A1* and is characterised by short-trunked dwarfism, kyphoscoliosis, enlarged joints, orofacial abnormalities, hearing loss, myopia, cataracts, lens luxation, blepharoptosis, abnormal vitreous and an increased risk of retinal detachment [21, 22]. Marshall syndrome is caused by an autosomal dominant mutation in *COL11A1* and shares many features with Stickler syndrome [15, 23, 26]. Additional findings may include short stature and more pronounced facial changes [15, 23, 25].

The Northern Inuit Dog (NID) breed is a relatively new breed developed in the 1980s in the United Kingdom, from dogs of unknown breeds imported from North America that were bred with the German Shepherd, Siberian Husky, Alaskan Malamute and possibly the Samoyed dog [27]. The intention was to breed a dog of wolf-like appearance that could be a family pet with an aptitude for work. The breed has since split into a number of similar breeds including NID, Tamaskan, Utonagan and British Timber dog and all are growing in popularity.

In this study, the clinical and histopathological findings and genetic basis of OSD in NID are described.

## Material and methods

### Ethics statement

All dogs used for this study were privately owned pet dogs and all were examined and treated at the owners’ request following informed and written owner consent. Buccal swab sampling, blood and ocular tissue collection were also performed following informed and written owner consent. Where DNA was obtained from blood, this sample was residual to blood drawn for diagnostic veterinary purposes, and was not drawn specifically for the purposes of research. Additionally, no *in vivo* experiments were undertaken. All clinical examinations and diagnostic investigations were conducted during the course of veterinary care and not specifically for research purposes. All sample collection was approved by the Animal Health Trust Ethics Committee (24-2018E).

### Clinical investigation

**Fig 1:**
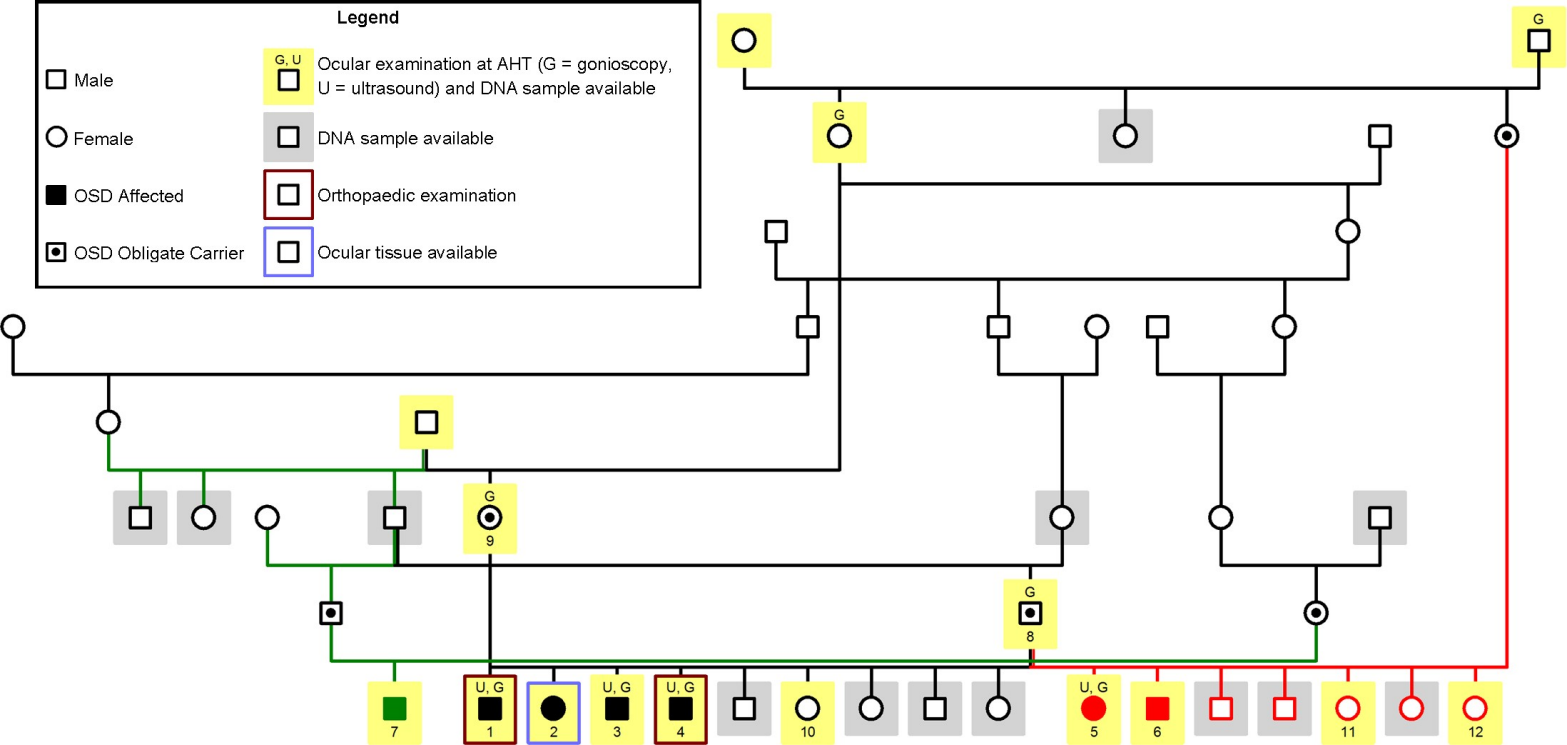
Pedigree of oculoskeletal dysplasia (OSD)-affected and related Northern Inuit Dogs (NID). OSD-affected and obligate carriers (sires and dams of OSD-affected NID), dogs that underwent ocular examination (including gonioscopy and ultrasound examinations where applicable), orthopaedic examination and the dog(s) from which DNA or ocular tissue were available, are indicated as in the legend. Coloured lines are used for better visualisation of breeding pairs. All OSD-affected NID are closely related and segregation of OSD is consistent with an autosomal recessive mode of inheritance.

Seven OSD-affected NID (Fig 1; Dogs 1–7) from three different litters were presented to the Comparative Ophthalmology unit of the Animal Health Trust (AHT) due to owner suspicion of visual impairment. Board-certified specialists in veterinary ophthalmology at the AHT examined and diagnosed all seven NID with OSD, with a clinical phenotype similar to that reported in the Labrador Retriever and Samoyed dog. Furthermore, 40 additional NID (8–47) that appeared OSD-unaffected, including littermates, parents, ancestors and dogs unrelated to the affected dogs, were recruited to form a clinical control cohort against which clinical findings from affected dogs could be compared. Clinical history and five-generation pedigrees were obtained from the owners of all 47 dogs.

All 47 dogs underwent ophthalmic and general physical examinations by first opinion veterinarians (MRCVS), board-certified veterinary ophthalmologists (CH, JO) and residents in veterinary ophthalmology (RS, RT); and two of the OSD-affected NID (Dogs 1 and 4) were also examined by board-certified veterinary orthopaedic specialists. Orthopaedic assessment consisted of visual analysis of gait and posture followed by palpation of bones, soft tissues and joints to assess musculature, swelling and pain. Finally, specific tests were performed including assessment of joint and bone instability; and digital skeletal radiography. A full ophthalmic examination was conducted, including vision assessment, Schirmer tear test (MSD Animal Health, Milton Keynes, Bucks, UK), slit-lamp biomicroscopy (Kowa SL-17, Torrance, California, USA), rebound tonometry (TonoVetTM iCare, FI-01510 Vantaa, Finland), and direct and indirect ophthalmoscopy (Keeler Professional, Windsor, Surrey, UK), including after dilation with 1% tropicamide (Minims, Bausch & Lomb, Kingston-upon-Thames, Surrey, UK). Gonioscopy was performed in five OSD-affected NID and in 21 OSD-unaffected NID; the iridocorneal angle was assessed as per the BVA/KC/ISDS Eye Scheme (https://www.bva.co.uk/Canine-Health-Schemes/Eye-scheme/). B-mode ocular ultrasound was performed in four OSD-affected NID (eight eyes) and one OSD-unaffected NID (one eye) (Easoate MyLabONE, 6–18MHz 30 mm linear probe, Imotek, Somersham, Cambs, UK). Ultrasound transmission gel (Aquasonic, Parker laboratories, USA) was used as a coupling medium. The ultrasound probe was orientated vertically in contact with the central cornea; an axial eye length was measured from the corneal endothelium to the retina–choroid–sclera complex. Gonioscopy and ultrasound were both performed after instillation of topical anaesthetic 0.5% proxymetacaine (Minims, Bausch & Lomb, Kingston-upon-Thames, Surrey, UK). Electroretinography was performed in two OSD-affected NID and one OSD-unaffected NID. Fundus photography by retinal camera was performed in three OSD-affected NID with mild lens opacity (RetCam Shuttle, Clarity, Macclesfield, Cheshire, UK).

**Fig 2:**
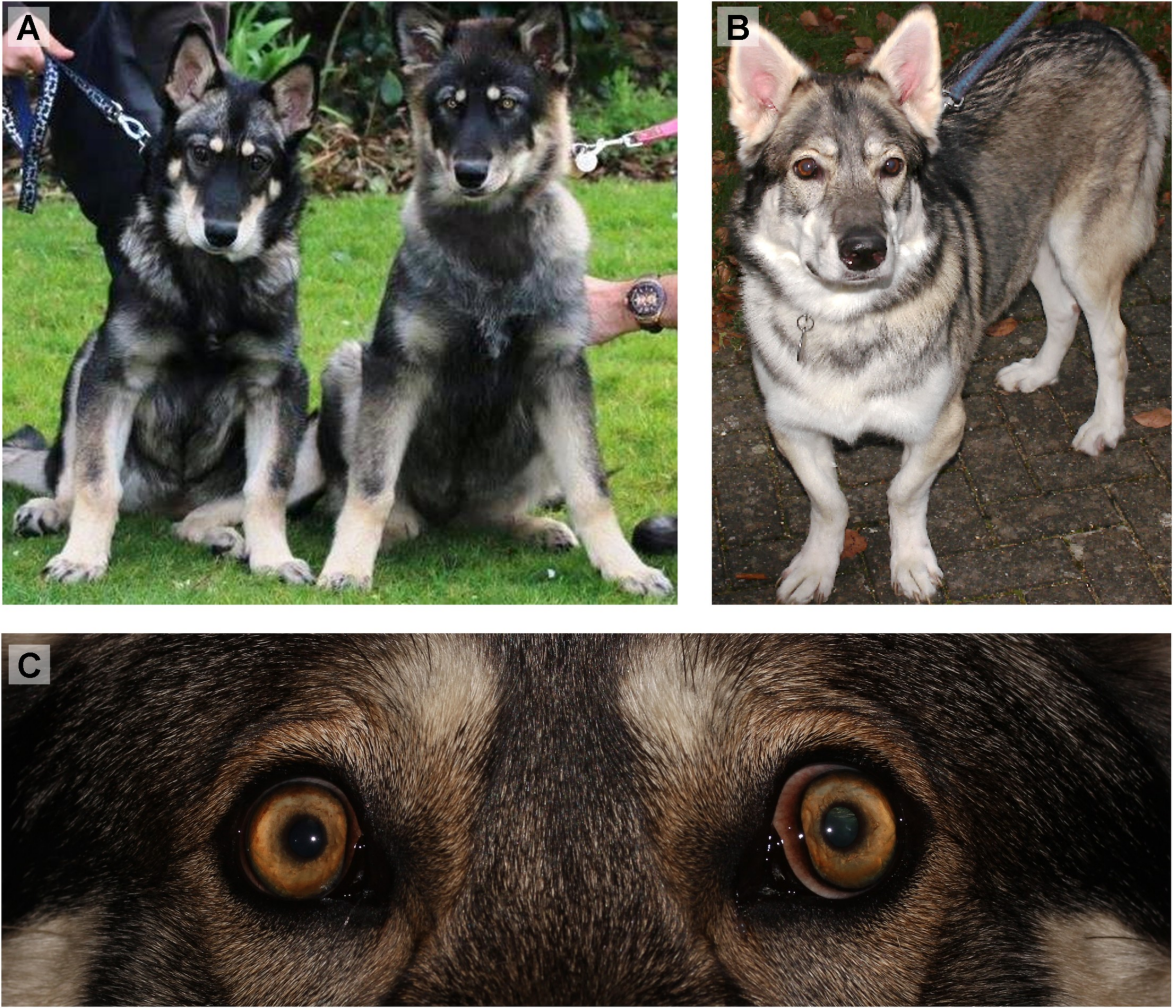
Appearance of oculoskeletal dysplasia (OSD)-affected Northern Inuit Dogs (NID). (A) OSD-affected three month old intact female NID (left, Dog 2) next to an unaffected three month old intact female littermate (right, Dog 11). (B) OSD-affected 3.5 year old neutered female NID (Dog 5). The OSD-affected dogs are shorter in stature, with obvious angular fore limb deformity i.e. varus elbows, valgus and short forelimbs. (C) Affected four month old male also presenting bilateral macroglobus (Dog 3).

**Fig 3:**
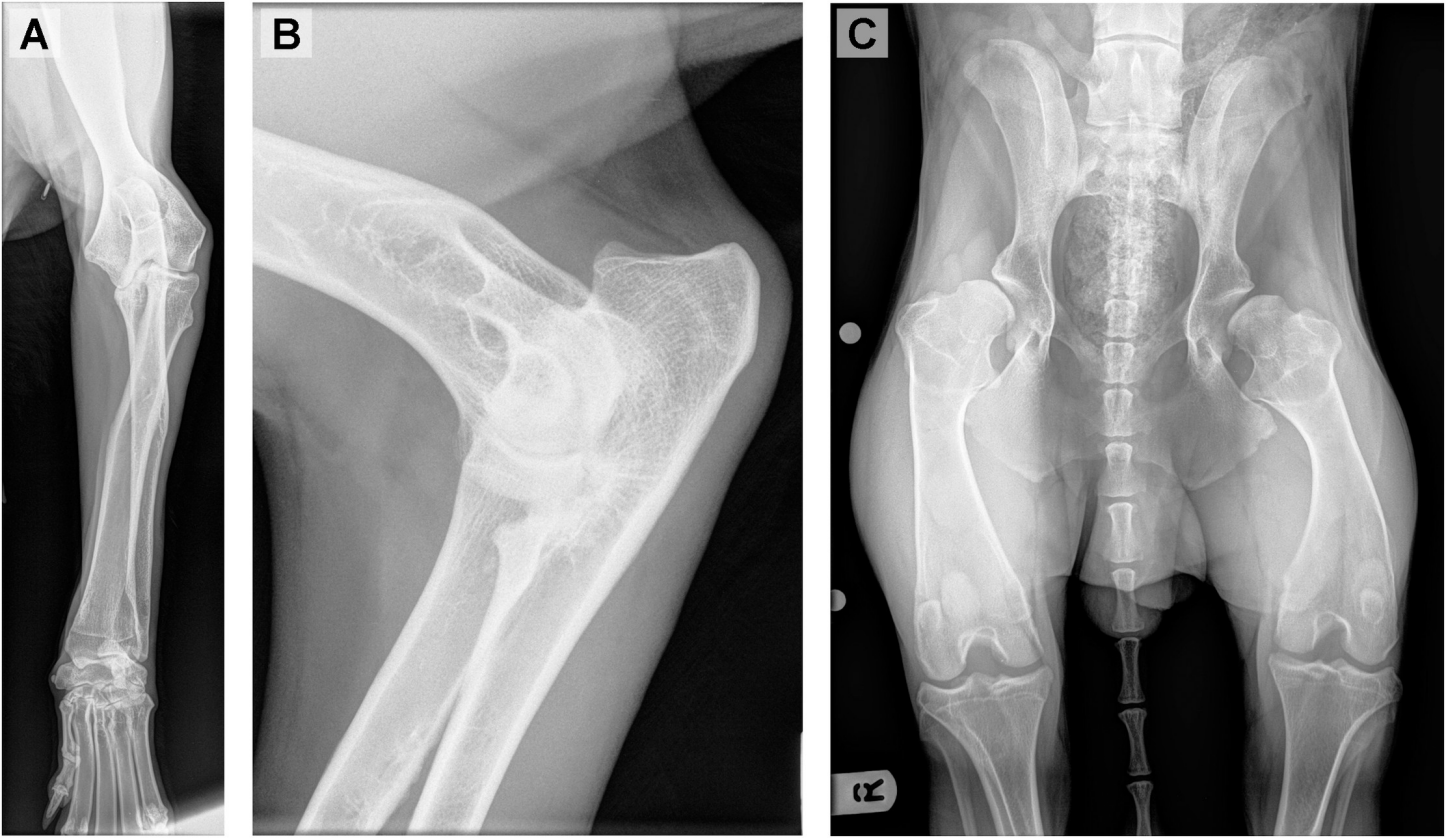
Skeletal defects associated with oculoskeletal dysplasia (OSD) in Northern Inuit Dogs (NID). (A-C) OSD-affected three month old NID (Dog 1). (A-B) Radiography of forelimbs revealed abnormally shortened long bones, antebrachial growth deformity, elbow dysplasia, incongruent elbow joints, radio-carpal subluxation and moderate osteoarthrosis. (C) Skeletal radiography of pelvis and hips revealed severe hip dysplasia consisting of subluxated and remodelled femoral heads and shallow acetabula, and mild osteoarthrosis. Long bones were also abnormally shortened.

**Fig 4:**
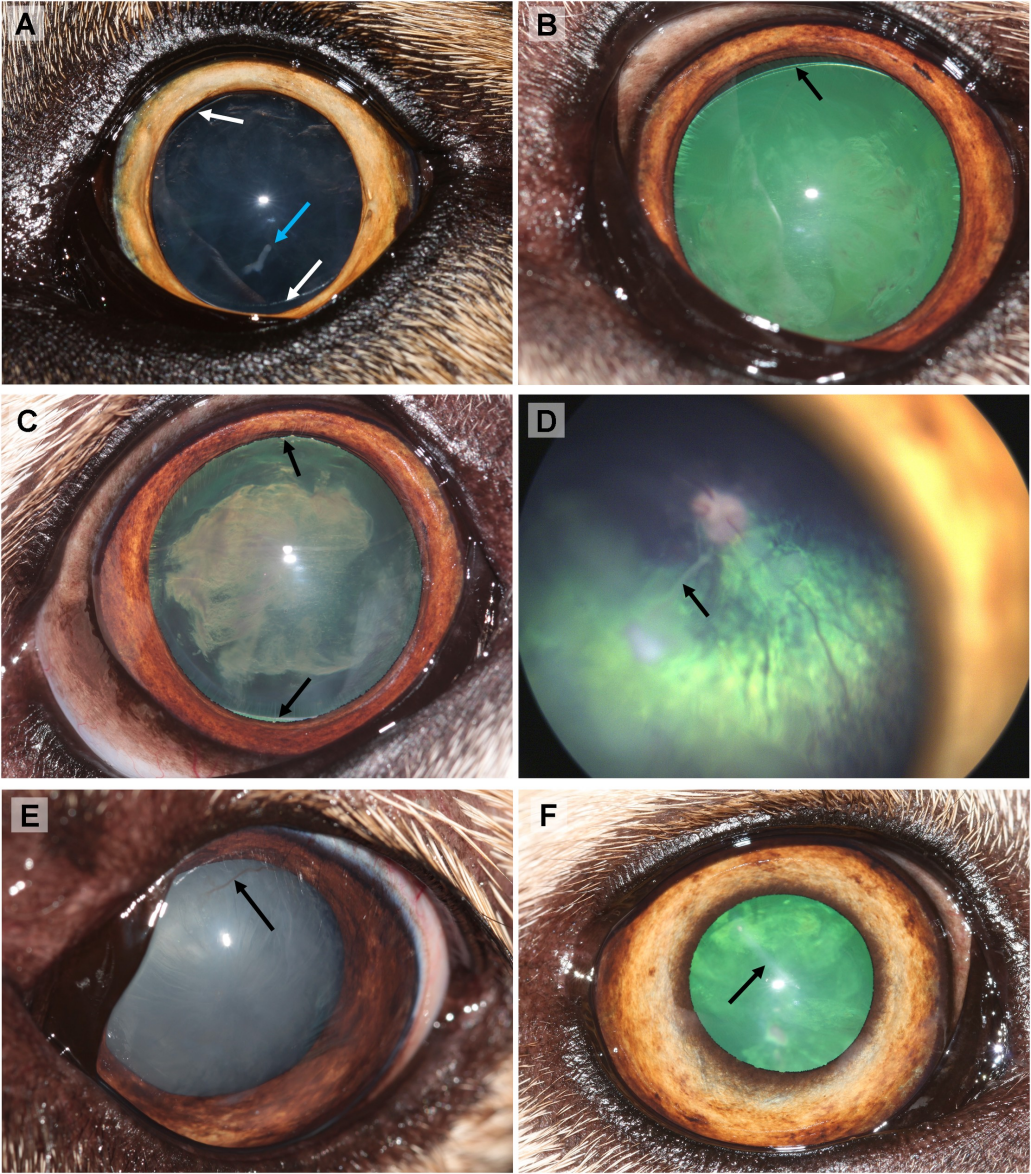
Ocular defects associated with oculoskeletal dysplasia (OSD) in Northern Inuit Dogs (NID). (A) An incipient cataract and lens coloboma (10–12 o’clock and 5–6 o’clock; white arrow) in the left eye. A vitreal tag (blue arrow) on the posterior lens capsule consistent with persistent hyaloid artery remnants is also present (Dog 3, four month old intact male) (B) An immature cataract and lens coloboma (11–1 o’clock; black arrow) in the left eye (Dog 6, 4.6 year old male intact male). (C) An advanced immature cataract and lens coloboma (11–1 o’clock and 5–6 o’clock; black arrow) in the right eye (Dog 6, 4.6 year old intact male). (D) RetCam photo of the retina in the right eye shows a lenticular-vitreal white strand consistent with persistent hyaloid artery remnants (black arrow). Retinal vessels appeared to be attenuated (Dog 5, 3.5 year old neutered female). (E) Corneal vascularisation associated with subtle fibrosis in the left eye (Dog 4, five month old neutered male). (F) Corneal fibrosis in the right eye (Dog 5, 3.5 year old neutered female).

OSD cases (n = 7) were defined as dogs displaying macroglobus, lens opacities, vitreo-retinopathies and disproportionate dwarfism (Figs 2–4).

### Sample collection

Buccal mucosal swabs or EDTA blood (residual to required veterinary procedures) were obtained from all seven OSD-affected NID, 116 OSD-unaffected NID and 545 dogs of OSD-unaffected non-NID breeds. The 116 OSD-unaffected NID comprised 40 NID that were clinically examined, 44 NID free of visual deficits as reported by their owners, and 32 NID with no owner’s report of signs relating to OSD. However, none of these dogs displayed obvious signs of skeletal dysplasia, and were therefore highly unlikely to be affected with OSD. Included in the group of 545 OSD-unaffected non-NID were breeds related to or from which NID were derived (45 Alaskan Malamute, 45 German Shepherd, 45 Samoyed dog, 45 Siberian husky and eight Utonagan) and 357 dogs of 122 OSD-unaffected non-NID breeds, all of unknown clinical status and collected for unrelated research projects.

Ocular tissue samples in the form of entire eye globes were obtained from one affected 18 month old NID (enucleated by the veterinarian as a part of required veterinary treatment for secondary glaucoma); from one OSD-unaffected 10 year old Golden Retriever (free from ocular abnormalities); and one four year old OSD-unaffected Irish Setter with unknown ocular clinical status, but not reported to be affected with OSD (both euthanased for unrelated medical reasons). One globe from the NID was preserved in formalin and sent for histopathological examination. The remaining globes were preserved in RNAlater (ThermoFisher Scientific) and stored at -70°C.

In addition to samples, whole genome sequence (WGS) data from 564 canids (comprising 554 dogs of 122 breeds, seven crossbreed dogs and three wolves) were also available. These included WGS from 112 dogs (none reportedly affected with OSD) sequenced for other projects, and 452 canids available through the Dog Biomedical Variant Database Consortium (DBVDC).

### Genetic investigation

Genomic DNA was extracted from buccal mucosal swabs using the QIAmp DNA Blood Midi Kit (Qiagen, West Sussex, UK) and from whole blood using from a Nucleon Genomic DNA Extraction Kit (Tepnel Life Sciences, Manchester, UK), both according to the manufacturer’s instructions. All primers and probes (S1 Table), unless otherwise specified, were designed using Primer3 [28] and purchased from Integrated DNA Technologies (IDT).

#### Screening of *drd1* and *drd2* mutations

The *drd1* mutation in *COL9A2* was assayed by PCR amplification using primers taken from the original publication (S1 Table and S1 Appendix) [5]; and the sizes of PCR products compared using agarose gel electrophoresis. The *drd2* mutation in *COL9A3* was assayed by amplified fragment length polymorphism (AFLP) analysis using a fluorescently-labelled and tailed third primer (S1 Table and S1 Appendix). Fragment sizes were detected using an ABI 3130xl Genetic Analyser (Applied Biosystems).

#### Whole genome sequencing and analysis

WGS of genomic DNA from two OSD-affected NID (dogs 1 and 4) was outsourced. The Welcome Trust Centre for Human Genetics generated a PCR-free library from one sample and paired-end sequencing was undertaken with a read length of 150bp on a HiSeq4000 (Illumina), resulting in ~15x genome coverage. Edinburgh Genomics generated a TruSeq Nano DNA library from the second sample and paired-end sequencing was undertaken with read lengths of 150bp on a HiSeqX (Illumina), resulting in ~30x genome coverage. Both datasets can be accessed via the European Nucleotide Archive (ENA accession number: PRJEB29115). Reads were aligned to CanFam3.1 using the Burrows-Wheeler Aligner [29] and variants called using GATK [30]. Integrative Genomics Viewer (IGV) was used to visualise and manually interrogate the WGS data using a candidate gene approach. Nine candidate genes that have been previously associated with animal and/or human OSD, or that are known to be highly expressed in the vitreous were selected (*COL2A1*, *COL5A2*, *COL9A1*, *COL9A2*, *COL9A3*, *COL11A1*, *COL11A2*, *OPTC* and *VIT*) [9–17, 21–24, 31–38]. Candidate genes were interrogated for single nucleotide polymorphisms (SNP), insertions and deletions in exons and introns, and structural variants by comparing WGS from the two OSD-affected NID with that of 26 dogs of different breeds (without OSD) and with the canine reference sequence (CanFam3.1). Breeds with hereditary retinal detachment and/or persistent hyaloid artery were excluded. Potential causal variants were further analysed using *in silico* bioinformatics tools to predict potential pathogenicity; SIFT (sorting intolerant from tolerant), PolyPhen and NCBI Mutation analyser to predict SNP pathogenicity, Splice Site Prediction by Neural Network [39–41], and Ensembl genome browser to assess nucleotide and amino acid conservation across 26 eutherian mammals of multiple species.

The genotype for the one remaining suggestive variant was determined in 1,232 dogs comprising 123 NID (seven OSD-affected and 116 OSD-unaffected) and 1,109 dogs of other breeds. The latter included 545 genotyped experimentally and 564 from existing WGS data (held at AHT or available through the DBVDC). A small cohort (seven OSD-affected NID and 26 OSD-unaffected NID) was initially genotyped by Sanger sequencing, with primers designed to flank the variant (S1 Table and S1 Appendix). Sequence traces were assembled, analysed and compared with the CanFam3.1 reference sequence using the Staden Package [42]. The entire cohort (123 NID and 545 non-NID) was subsequently genotyped using an allelic discrimination assay. Reactions were carried out in 8 μL volumes consisting of 4 μL Kapa probe fast (Kapa Biosystems), 0.1 μM each primer and 0.2 µM each probe (S1 Table) and 2 μL genomic DNA. Cycling parameters were 25°C for 30 sec, 95°C for 3 min, 40 cycles of 95°C for 3 sec and 60°C for 10 sec, and finally 25°C for 30 sec. PCR amplification and allelic discrimination analysis were carried out on an ABI StepOne Plus with the STEPONE Software v 2.2.2 (Life Technologies).

#### Quantitative reverse-transcription PCR

Total RNA was extracted using an RNeasy Mini Kit (Qiagen) and reverse transcribed to produce cDNA using the Quantitect reverse transcription kit (Qiagen), which includes a dsDNA removal step using DNA Wipeout buffer, both according to the manufacturer’s instructions. PrimeTime Probe-based assays targeting *COL9A3* (exons 28–30) and TATA-binding protein (*TBP*, a house-keeping gene for normalisation of fluorescent signals*)* (exons 3–4) were designed using the PrimerQuest design tool (Integrated DNA Technologies) (S1 Table). Primers flanking each of these targets were used to generate PCR amplicons, which in turn were used as templates to generate standard curves. Quantitative PCR (qPCR) was carried out on an ABI StepOne Plus (Life Technologies) in 13 μL reactions, comprising 1x Luna Universal Probe qPCR Master Mix (NEB), 1x *COL9A3*/*TBP* assay, 5% DMSO (in *COL9A3* reaction only) and 3 μL DNA template. Thermal cycling conditions were as follows: 50°C for 2 min, 95°C for 10 min, followed by 40 cycles of 95°C for 10 sec and 62°C for 30 sec. Reaction efficiencies were calculated using a six-point 5x serial dilution to create a standard curve. *COL9A3* and *TBP* reaction efficiencies were estimated at 84.2% and 93.7% respectively, with R^2^ values both >0.995. Reactions were performed in triplicate, C_T_ values of *COL9A3* were normalised to those of *TBP*, and comparisons between the OSD-affected NID and OSD-unaffected non-NID were performed with the ΔΔC_T_ method [43]. The final qPCR products were also analysed by agarose gel electrophoresis to confirm amplification of cDNA and not gDNA (S2 Appendix).

## Results

### OSD-affected NID presented with skeletal and ocular deformities

All study dogs were purebred NID residing in the UK. Based on pedigree data provided by the owners, an extensive pedigree for related NID, especially those closely related to the OSD-affected NID, was established (Fig 1). Dogs 1 to 4 (three males and one female) were littermates and presented to the AHT at three to four months of age with a history of variable degrees of visual impairment. Dogs 5 (female) and 6 (male) were from a second litter from the same sire (Dog 8) and presented with a similar clinical history. Dog 7 (male) was from a third litter and his sire was a half-brother of Dog 8. Pedigree analysis revealed that OSD in NID is inherited as an autosomal recessive trait.

Phenotypically, all affected NID (Dogs 1–7) displayed skeletal dysplasia with short-limbed dwarfism and angular limb deformities (Fig 2). Orthopaedic assessments of Dogs 1 and 4 had been undertaken at AHT and another referral institution respectively, but with broadly similar results consisting of disproportionate short-limbed dwarfism (short appendages with a normal axial skeleton) and skeletal dysplasia; skeletal radiographs were available from Dog 1 taken at one year of age (Fig 3). Pre-anaesthetic haematology and biochemistry on Dog 1 did not reveal any significant abnormalities (Table 1). Dog 6 had a history of hearing problems, observed by the owner, but it is unknown if these are related to OSD (Table 1).

**Table 1:** Clinical investigation of seven OSD affected NID.

**Dog No**	1	2	3	4	5	6	7
**Sex**^**1**^	M	F	M	M	FN	MN	MN
**Age/months**	3	4	4	5	42	55	48
**Initial vision assessment**	OD: visual	OD: blindness	OU: visual	OU: blindness	OD: visual	OU: visual impairment	OU: visual impairment
	OS: blindness	OS: visual impairment			OS: visual impairment		
**Macroglobus**	+	+	+	+	+	+	+
**Cataract**	Immature cortical	Immature cortical	Incipient cortical	Immature cortical associated with iris hyperpigmentation	Immature cortical	Immature cortical	Mature
**Lens coloboma**	-	-	+	-	+	+	-
**Microphakia**	+	-	+	+	+	+	N/A
**Vitreopathy**	+	+	+	+	+*	+	+
**PHAR**^**2**^	+	+	+	-	+	+	N/A
**Retinal detachment**	+*	+	-	+	+*	-	+
**Retinal degeneration**	+*	N/A	+	N/A	+*	+	N/A
**ONH**^**3**^	pale*	N/A	pale	N/A	-*	pale	N/A
**Tapetal hypoplasia**	-*	N/A	+	N/A	-*	-	N/A
**Concurrent ocular findings**	N/A	N/A	iris nevus*, persistent pupillary membrane*	vertical—wandering nystagmus	corneal fibrosis*	multiple hyper-reflective focal tapetal lesions	N/A
**Osteochondrodysplastic (disproportionate) dwarfism phenotype**	+	+	+	+	+	+	+
**Hearing difficulties**	NAD	NAD	NAD	NAD	NAD	suspected by the owner	NAD
**Gonioscopy**	OD: NAD	OU: NAD	OU: NAD	OU: NAD	OU: NAD	OU: NAD	N/A
	OS: narrow iridocorneal angle and mild goniodysgenesis						
**Further investigation**^**4**^	ERG: OD good rod/cones response, OS no rod/cones response	ERG: N/A	ERG: N/A	ERG: OU: no rod/cones response	ERG: N/A	ERG: N/A	ERG: N/A
	OUS: OU: macroglobus (the axial length: OD 27.2mm; OS 28.5mm), microphakia, hyperechoic lens, vitreal degeneration; OS retinal detachment	OUS: N/A	OUS: OU macroglobus (the axial length: OD 29mm; OS 28.8mm), microphakia, hyperechoic lens, vitreal degeneration	OUS: OU: macroglobus (the axial length: OD 28.9mm; OS 29mm), hyperechoic lens, microphakia, posterior subluxation, vitreal degeneration, retinal detachment	OUS: OU: macroglobus (the axial length: OD 27.1mm; OS 26.4mm); OD hyperechoic lens, remnant of persistent hyaloid artery; OS hyperechoic lens, vitreal degeneration, suspected remnant of persistent hyaloid artery, a focal prominent thickening of the retina adjacent to the optic nerve head—suspected retinal detachment	OUS: N/A	OUS: OU: hyperechoic lens, vitreal opacities (interpreted as degeneration), retinal detachment, no measurements taken
	SOAR: front and hind angular limb deformities, elbow dysplasia, severe hip dysplasia, chondrodystrophic conformation	SOAR: N/A	SOAR: N/A	SOAR: ambulatory without gait asymmetry. Both forelimbs were symmetrically markedly shortened in length, with radial procurvartum and carpal valgus, consistent with osteochondrodysplatic dwarfism, or skeletal dysplasia. The hind limbs were also shorter but significant deformity was not present.	SOAR: N/A	SOAR: N/A	SOAR: N/A
	Haematology: mild eosinophilia, 2.04x10^9^/L (reference value 0.20–1.20 x 10^9^/L); Biochemistry: normal	Blood test: N/A	Blood test: N/A	Blood test: N/A	Blood test: N/A	Blood test: N/A	Blood test: N/A
**Follow up**^**5**^	24.6 months	20.9 months	23.2 months	21.4 months	33.5 months	0.9 month	47.6 months
	OU: LIU	OU: LIU, suspected secondary glaucoma	OU: progression of the cataract to immature, visual impairment	OU: progression of the cataract to mature, posterior lens subluxation, LIU	OU: progression of cataract (OS mature); posterior lens subluxation, LIU	OU: worsening of vision due to suspected progression of the cataract	OU: blindness
	OD—progression of the cataract, eye still visual						
	OS progression of the cataract to mature, secondary glaucoma, retinal vessel attenuation	OS: blindness		OS: concurrent ocular finding: corneal vascularisation associated with subtle fibrosis	OS: blindness		
**Treatment**^**6**^	OU: Acular (ketorolac maleonate), BID	OU: Enucleation	OU: Acular (ketorolac maleonate, BID	OU: Yellox (bromfenac), BID, Latanoprost BID	OU: Yellox (bromfenac) BID	OU: Acular (ketorolac maleonate) BID	N/A
	OS: Cosopt (dorzolamide/timolol), BID						

OSD—oculoskeletal dysplasia, NID—Northern Inuit Dog; OD—right eye, OS—left eye, OU—both eyes; + = positive,— = negative; unless marked with *, lesions were present in both eyes; NAD—nothing abnormal detected, N/A—not applicable

^1^M –male, MN–male neutered, FN–female neutered

^2^PHAR–persistent hyaloid artery remnants

^3^ONH–optic nerve head

^4^ERG–electroretinography, OUS–ocular ultrasound, SOAR—specialist orthopaedic assessment/radiography

^5^Follow up—period between first evidence of eye problem and last seen by veterinary surgeon; LIU–lens induced uveitis

^6^BID–twice daily

Dogs 1–7 were all subjected to thorough ocular examination (Table 1). Findings included combination of reduced menace response consistent clinically with visual impairment, blindness (absent menace response), immature to hypermature cataract, lens coloboma, microphakia, vitreopathy, persistent hyaloid artery remnants on the posterior lens capsule, retinal degeneration and retinal detachment (Fig 4A–4D). Dog 6 had a bilateral hyper-reflective focal tapetal lesion. Gonioscopy revealed no abnormalities in four out of five tested dogs in both eyes; Dog 1 had a narrow iridocorneal angle (ICA) and mild goniodysgenesis (less than 25% of the iridocorneal angle was affected) in one eye with secondary glaucoma due to lens induced uveitis; the other eye had normal anatomy of the ICA. Electroretinography performed in two NID revealed bilateral absence of rod/cone response in Dog 4 and in the left eye of Dog 1. Ocular ultrasound was performed in four out of seven OSD-affected dogs (Table 1). The axial length of the eight globes (four dogs) ranged from 26.42 mm to 29.12 mm, compared to 21.53 mm in the right eye of one OSD-unaffected NID and with published ocular biometric data obtained by computer tomography that the horizontal / sagittal eye length was 22.23/ 22.57 mm respectively in the large breed group [44]. Concurrent clinical ophthalmic findings included unilateral dorsal corneal superficial vascularisation (Dog 4) (Fig 4E), linear axial corneal superficial fibrosis (Dog 5 unilateral) (Fig 4F), unilateral iris nevus (Dog 3), unilateral persistent pupillary membrane (Dog 3) and bilateral vertical nystagmus (Dog 4).

**Fig 5:**
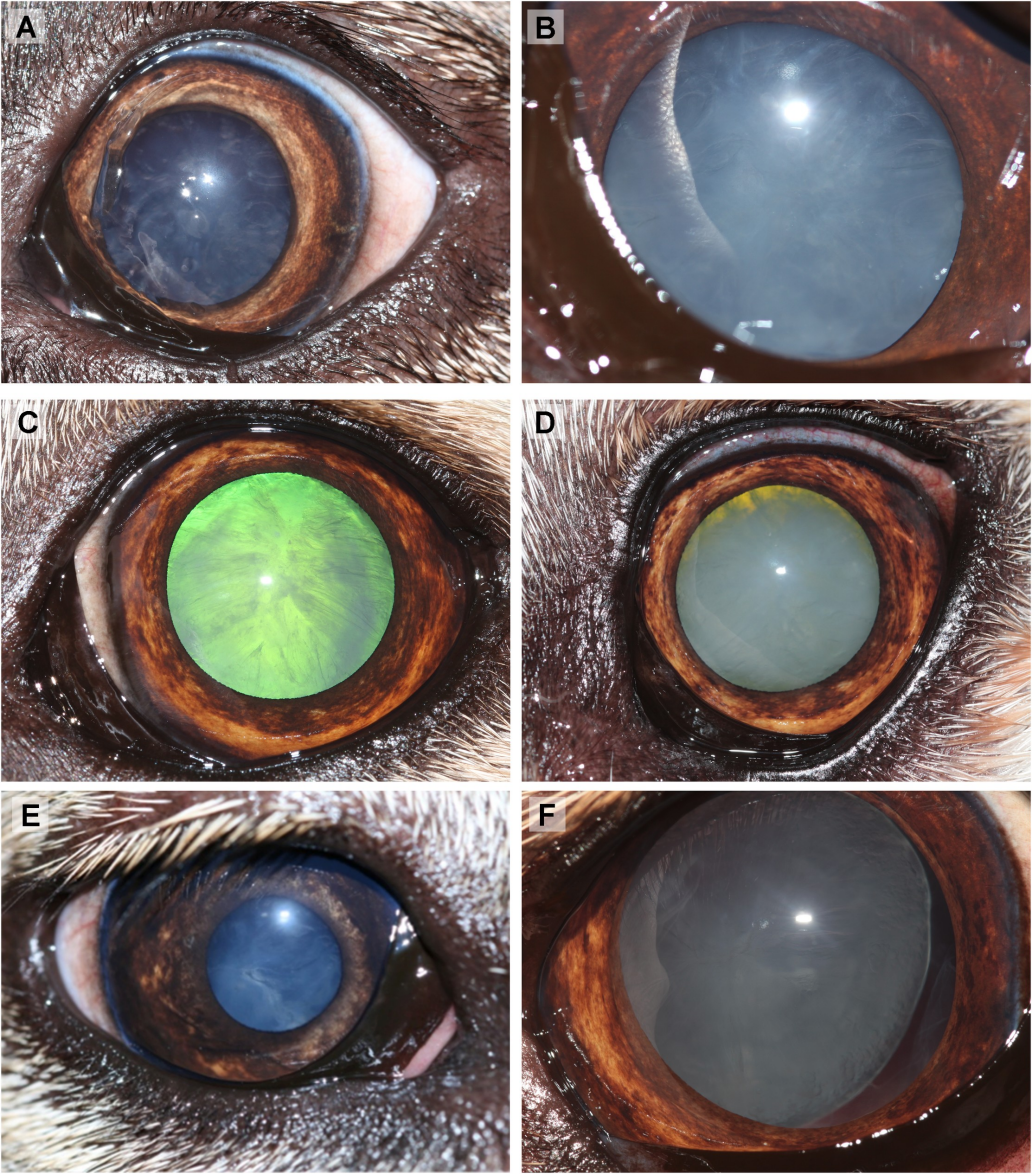
Progression of ocular lesions in oculoskeletal dysplasia (OSD)-affected Northern Inuit Dogs NID. In the left eye of Dog 1, immature cataract (A) progression associated with lens induced uveitis presented as iridal hyperpigmentation (B). In the left eye of Dog 5, an immature cataract (C) progressed to an advanced immature cataract (D). In the right eye of Dog 4, immature cataract (E) progression was associated with lens induced uveitis, and posterior lens subluxation was also observed (F).

Follow-up was possible in all dogs, with a median follow-up period of 23.2 months (ranging from 0.9 month to 47.6 months) (Table 1). Ocular lesions progressed in most of the OSD-affected NID to a combination of advanced cataracts (five dogs/10 eyes) associated with visual impairment (two out of 5 dogs/4 eyes), lens induced uveitis (four dogs/eight eyes), posterior lens subluxation (two dogs/four eyes), secondary glaucoma (two dogs/three eyes), and blindness (three dogs/four eyes) (Fig 5). Ocular pathology of one globe (Dog 2) (Emma Scurrell, Cytopath Ltd., UK) revealed a hydrophthalmic globe with lens luxation, anterior, equatorial and posterior cortical cataract, vitreal syneresis, chronic rhegmatogenous and retinal detachment. Anterior to posterior dimension of the lens was 5 mm and the dorso-ventral dimension was 12 mm. There was diffuse inner nerve fibre atrophy with variable ganglion cell loss and diffuse atrophy of the photoreceptor processes (consistent with detachment). Retinal dysplasia (rosettes) was not evident within the detached and atrophied retina. A presumptive diagnosis of a vitreoretinopathy was made.

### Clinical control cohort

An additional 40 NID (24 female and 16 male) with a median age of 4.7 months (ranging from 1.2 to 11.8 months), including 16 closely related to the OSD-affected NID, underwent ophthalmic and physical examinations. Gonioscopy was performed in 21 dogs: seven dogs had a normal anatomy of ICA, three dogs were mildly affected (goniodysgenesis involved less than 25% of ICA), six dogs were moderately affected (goniodysgenesis involved more than 25% to 75% of ICA) and one dog was severely affected (goniodysgenesis involved more than 75% of ICA). None of those with affected ICA displayed signs of glaucoma at the time of examination. The remaining four dogs presented with a completely closed ICA; three of them were clinically diagnosed with glaucoma. In two dogs/three eyes histopathological examination revealed primary closed angle glaucoma. None of the NID in the clinical control cohort (n = 40) displayed signs consistent with OSD. Concurrent ocular findings in those 40 dogs were persistent pupillary membrane and nuclear sclerosis (one dog), and a focal area of tapetal hyper-reflectivity consistent with a retinal scar (two dogs). Two dogs, aged 9 and 10 years, also had an incipient senile cataract. Four dogs, aged five months to four years, had mild-moderate anterior vitreal degeneration but no other ocular abnormalities.

### Exclusion of *drd1* and *drd2*

All seven OSD-affected NID and four OSD-unaffected NID were genotyped for the mutations previously associated with OSD in the Samoyed dog (*drd2*, *COL9A2*) and the Labrador Retriever (*drd1*, *COL9A3*). All 11 dogs were homozygous for the wildtype alleles at both loci; *drd1* and *drd2* were therefore excluded as causal for OSD in the NID.

### Candidate causal variant identified through WGS

**Fig 6:**
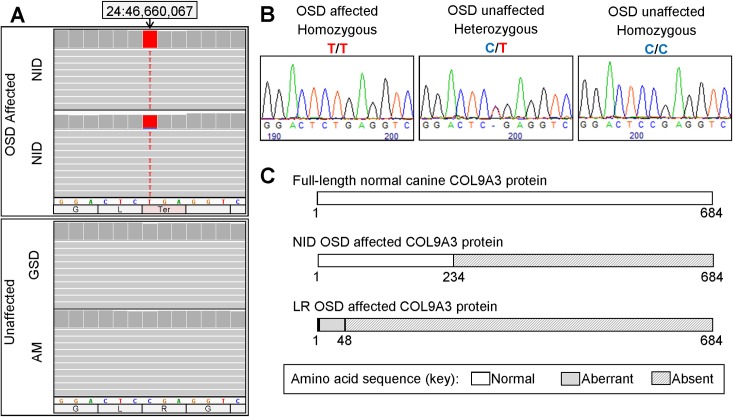
Nonsense SNP identified in *COL9A3*. *COL9A3*c.700C>T (at chr24:46,660,067 in CanFam3.1) was identified by interrogation of WGS using IGV (A) and confirmed by Sanger sequencing (B). Oculoskeletal dysplasia (OSD)-affected NID were homozygous for the variant (T/T); obligate carrier Northern Inuit Dogs (NID) were heterozygous (C/T); OSD-unaffected NID were either heterozygous or homozygous for the wildtype allele (C/C); and all non-NID breeds were homozygous for the wildtype allele (C/C). The variant is expected to change codon 234 from CGA (encoding Arginine) to TGA (a termination codon), resulting in a protein that is significantly truncated (C). The *COL9A3* frameshift variant reportedly associated with OSD in Labrador Retriever is predicted to disrupt the entire protein, resulting in 48 aberrant amino acids before a premature termination codon.

Manual interrogation of the exons and introns of nine candidate genes using IGV (Integrative Genomics Viewer [45]) revealed only one variant that was homozygous in two OSD-affected NID but not in 26 OSD-unaffected non-NID. This was a *C/T* SNP at chr24:46,660,067 (CanFam3.1), in exon 14 of the *COL9A3* gene, and was predicted to be pathogenic (Fig 6). Comparison of the human Ensembl *COL9A3* transcript (ENST00000343916.7; 2485 bp, 684 amino acids and 32 exons) with the canine Ensembl transcript (ENSCAFT00000020179.3; 1869 bp, 623 amino acids and 31 exons) suggested exons 15 and 16 of the canine transcript might be inaccurate, and exon 32 is absent from the annotation. Messenger RNA sequence generated by Goldstein et al (NCBI accession number GU075882; 2682 bp, 684 amino acids and 32 exons) confirmed that the canine *COL9A3* gene is very similar to the human gene [5]. Based on this, the *COL9A3* variant is predicted to be a nonsense SNP (c.700*C>T*) within a collagen triple helix repeat region (COL3), and to result in a premature termination codon and a protein product truncated by 451 amino acids (p.Arg234Ter) (Fig 6). The nucleotide and amino acid affected by the SNP are conserved in 24/26 eutherian mammals.

### *COL9A3* variant segregates with OSD in NID

The *COL9A3* variant was assessed by Sanger sequencing in an initial cohort of seven OSD-affected and 26 OSD-unaffected NID (including 19 that had undergone ophthalmological examination and had normal, healthy eyes). All seven OSD-affected NID were homozygous for the variant allele (*T/T*), while 14 OSD-unaffected NID were heterozygous (*C/T*) and 12 OSD-unaffected NID were homozygous for the wildtype allele (*C/C*). An allelic discrimination assay was subsequently developed for all genotyping, including 90 additional OSD-unaffected NID, all of which were either heterozygous (*C/T*, n = 17) or homozygous for the wildtype allele (*C/C*, n = 73). Overall, seven OSD-affected NID were homozygous for the variant allele and 116 OSD-unaffected NID were either heterozygous (n = 31) or homozygous for the wildtype allele (n = 85), confirming that the variant is statistically associated with OSD (Fisher’s exact P-value = 1.4x10^-11^). Pedigree analysis and the observation that only dogs homozygous for the *COL9A3* mutation are clinically OSD-affected NID are consistent with the disease having an autosomal recessive mode of inheritance with complete penetrance. In order to estimate the variant allele frequency 56 NID were identified from the entire NID cohort that were not related at the parent level. Nine were heterozygous and 47 were homozygous for the wildtype allele, resulting in a variant allele frequency of 0.08.

All 1,109 non-NID dogs (545 genotyped experimentally and 564 ascertained from WGS data) were homozygous for the wildtype allele (*C/C*), suggesting this variant is likely to be private to the NID breed. Included in the 545 dogs genotyped experimentally are 45 each of the four breeds that were reportedly used to create the NID breed; German Shepherd, Alaskan Malamute, Siberian Husky and Samoyed dog, as well as eight of the NID-like Utonagan breed.

### *COL9A3* mRNA transcript levels may not be affected

**Fig 7:**
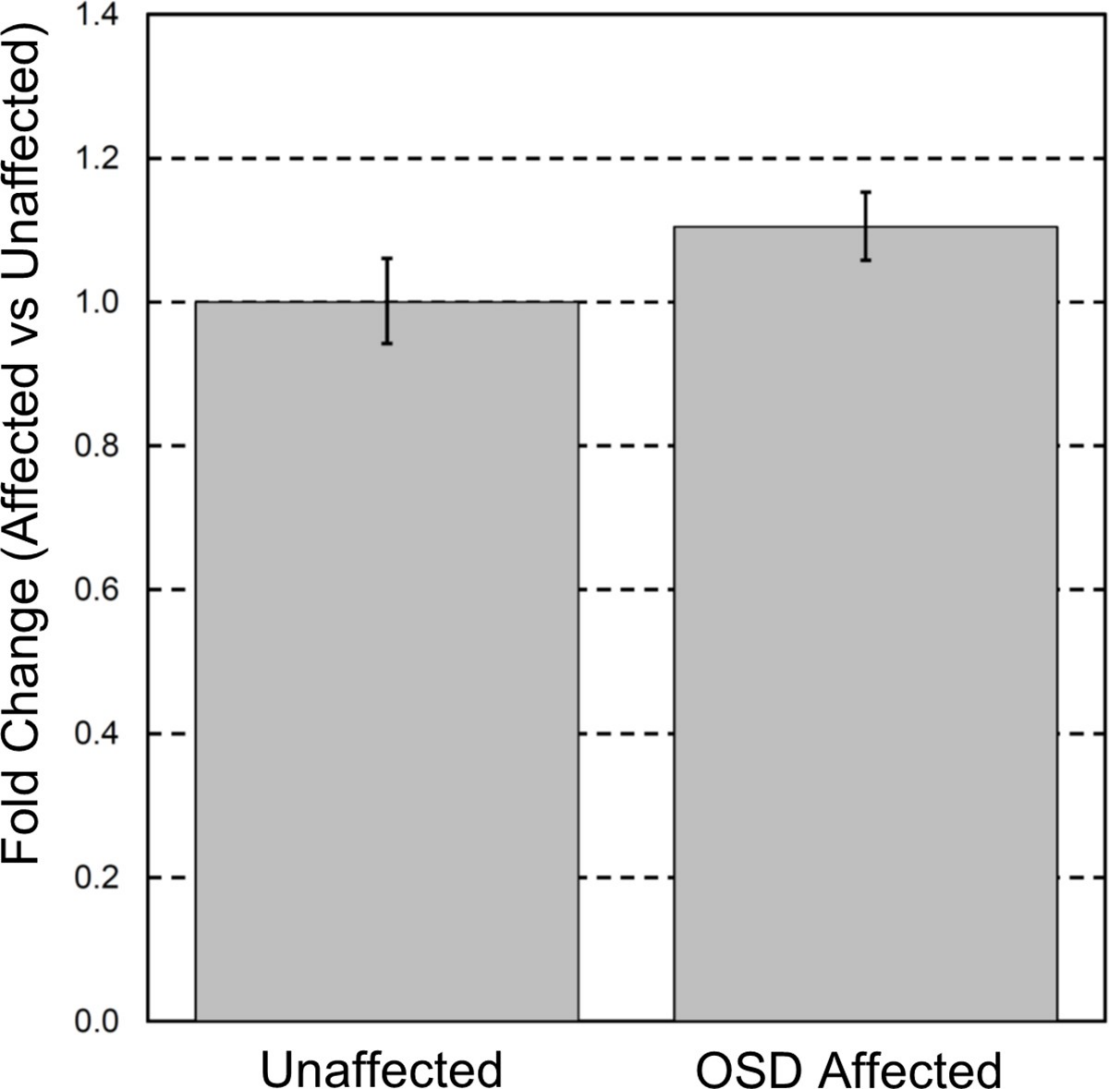
Effect of *COL9A3* variant on mRNA. Quantitative RT-PCR: Expression of *COL9A3* transcript is not significantly altered {Fold change = +1.1; measured by qRT-PCR in an oculoskeletal (OSD)-affected Northern Inuit Dog (NID) and unaffected non-NID}. Error bars show the standard deviation of technical triplicates.

Relative quantitative RT-PCR was carried out using cDNA generated from retinal tissue from an OSD-affected NID (Dog 2) and OSD-unaffected non-NID, comparing *COL9A3* with the housekeeping gene *TBP*. The OSD-affected NID sample showed a 10% increase in *COL9A3* expression (Fold change = +1.1) (Fig 7). However, the reaction efficiency of 84.2% suggests that this assay is not optimal. Agarose gel electrophoresis of the final products confirmed amplification of mRNA and not gDNA (S2 Appendix). The suboptimal reaction efficiency could therefore possibly be due to the use of suboptimal primers. Another assay in *COL9A3*, was performed but the reaction efficiency was worse. The entire *COL9A3* gene has a GC content much higher than *TBP* approximately 70% and 40% respectively, which will have contributed to the difficulty in designing optimal *COL9A3* primers. Nevertheless, whilst these results are inconclusive, they do suggest that nonsense-mediated decay of the mutated *COL9A3* transcript may not contribute to pathogenesis, and if this is the case a truncated protein product is expected to be produced.

## Discussion

Inherited OSD, described in the Labrador Retriever and Samoyed dog, had not been previously identified in NID or any other breeds. The clinical phenotype of OSD-affected NID in the current cohort appears to be similar to the Labrador Retriever and Samoyed dog [1–4, 6, 7]. Moderate corneal vascularisation or pigment swirling and opacification were observed in some affected Labrador Retrievers with OSD and are also a feature of Stickler syndrome in humans [1, 4, 8, 46, 47]. Corneal superficial vascularisation and/or fibrosis of unknown origin were also observed in two OSD-affected NID.

Several differences were noted between the OSD-affected Labrador Retriever and Samoyed dogs and OSD-affected NID. Unlike Labrador Retrievers, OSD-affected NID did not present with corneal pigment swirling or retinal dysplasia (folds or rosettes), and they rarely presented with alteration in optic nerve colour and size or with tapetal hypoplasia [1–5]. Conversely, microphakia, lens coloboma and retinal degeneration observed in OSD-affected NID were not reported in the other two breeds [1–7].

Blair et al. reported that retinal detachment in OSD-affected Labrador Retrievers might occur due to retinal tears secondary to vitreoretinal traction caused by vitreous abnormalities, which is consistent with reports of OSD in humans [4, 8, 14, 48, 49]. As vitreal syneresis was also present in the current study, the same pathogenesis of complete retinal detachment is hypothesized. The NID globe with retinal detachment revealed no signs of retinal folds or rosettes.

Aroch et al. described brachygnathia and haematological abnormalities (marked eosinophilia, eosinophilic bands, absence of Barr bodies, lymphocytosis and neutrophilia) in the OSD-affected Samoyed dog [7]. In contrast, orofacial abnormalities or hearing difficulties in the Labrador Retriever or Samoyed dog were not reported in subsequent studies, although specific examinations were not conducted [1–5]. Most OSD-affected NID did not display any signs of hearing impairment, apart from Dog 6, although specific quantitative hearing tests were not undertaken. Similarly, neither cranial imaging nor rhinolaryngological examination was performed in the NID OSD-affected dogs, and mild orofacial abnormalities cannot be completely excluded. A blood work up was not routinely performed in cases in the present study, although one tested dog (Dog 1) showed mild eosinophilia.

The present study reports, for the first time, the progression of ocular signs associated with OSD. However, it is unclear whether this progression is primarily due to OSD or is secondary to maturing cataracts. It is possible that phacoemulsification during the early stages of disease could prevent lens induced uveitis and associated complications; on the other hand it could have a similar effect as in humans, where it can increase risk of retinal detachment or glaucoma and a prophylactic retinopexy is usually performed [50].

A small number of skeletally normal Labrador Retrievers heterozygous for the OSD mutation presented with focal or geographic retinal dysplasia [5, 6]. In contrast, all skeletally normal NID, including 14 that were subsequently identified as heterozygotes, were free of ocular defects typical of OSD. Goniodysgenesis was observed in 14 OSD-unaffected NID, three of which later developed glaucoma. Unlike human patients with STL, in whom an abnormal ICA has been reported [25], five out of six OSD-affected NID whose ICA was assessed (either by gonioscopy or histopathologically) had normal ICAs. Interestingly, goniodysgenesis is associated with primary closed angle glaucoma in three of the NID founding breeds—the Siberian Husky, Alaskan Malamute and Samoyed dog [51]. The significance of these findings in NID is unknown and further studies are warranted.

Clinical macroglobus presented in all affected NID was confirmed by ocular ultrasound in five dogs. Enlarged globes were confirmed in all OSD-affected NID (Table 1) when compared with one OSD-unaffected NID (with the axial length of 21.53 mm). OSD-affected NID globes were also compared with ocular biometric data obtained by computer tomography that the horizontal/sagittal eye length was 22.23/22.57mm respectively in the large breed group [44]. Interestingly the axial length of the right globe, measured in six Samoyed dogs, ranged from 19 to 26mm [7].

A SNP in exon 14 of *COL9A3* (collagen type IX Alpha 3 Chain; c.700C>T; g.chr24:46,660,067) was identified, which is predicted to result in a premature termination codon (p.R234Ter), and a truncated protein containing only 234 amino acids, instead of 684 amino acids (Fig 6). Collagens are the most abundant proteins of the extracellular matrix (ECM) and have a range of functional roles, including contributing to the stability and structural integrity of organs and tissues [52]. Type IX collagen is a FACIT collagen and regularly aligns along the surface of types II and XI fibrils [52–59], and functions to shield type II from exposure to the surface of the vitreous fibrils. Collagen fibrils lose type IX collagen during the normal aging process of vitreous, which predisposes the fibrils to fusion, and this underlies the liquefaction and weakening of vitreoretinal adhesion [17, 60]. This in turn leads to the detachment of the vitreous or the development of retinal tears or holes [17]. Similarly, in OSD-affected NID, the variant in *COL9A3* most likely disrupts the function of collagen IX, which presents clinically as vitreoretinopathy and skeletal dysplasia.

*COL9A3*_c.700C>T_ occurs approximately halfway through the COL3 domain, resulting in the loss of half of the COL3 domain and the COL1-2 and NC1-3 domains. Both of the OSD mutations previously reported, in the Labrador Retriever and Samoyed dog, also affect the COL3 domain of their respective genes, which could explain similar clinical signs observed in all three breeds [5].

The Labrador Retriever mutation, occurring in exon 1 and affecting almost the entire protein (Fig 6), leads to lack of mRNA expression [5], whereas there was no significant difference between the expression of *COL9A3* mRNA between the OSD-affected NID and OSD-unaffected non-NID (Fig 7). However, it is important to note that biological replicates of affected and OSD-unaffected tissue were not available, and therefore it has not excluded the possibility that the expression observed here is not representative of disease-associated expression in general. In addition, the affected NID was only 18 months old at the time of enucleation, whereas the OSD-unaffected non-NID was 10 years old when the tissue was harvested. Age-matched control tissue was not available for this study. Global transcriptomic studies in rats have revealed that *COL9A3* expression is highest in the brain (the most similar tissue to retina for which data is available) of juvenile rats. In adolescent rats the expression level halves, and in adult rats it halves again [61] (viewed on NCBI, Gene ID 362285). Similarly, the Mouse ENCODE project found that the highest *COL9A3* expression levels were seen in the E14.5 limb, followed by E14 central nervous system (CNS, at 20% of the limb levels) and finally in the adult brain (at 25% of the E14 CNS level) [62] (viewed on NCBI, Gene ID:12841). Assuming that a similar pattern would be observed in the canine retina (such data are not available to our knowledge), the *COL9A3* expression that was observed in the OSD-affected NID could be interpreted as abnormal, in that in a healthy adolescent dog it would be expected to be significantly higher than in a geriatric dog. Nevertheless, due to the limitations of our study (lack of biological replicate and age-matched healthy tissue), no conclusions can be made, and this is only speculation. For similar reasons attempts to investigate protein expression by western blot resulted in ambiguous results from which no conclusions could have been drawn (S2 Appendix).

An autosomal recessive loss of function mutation in exon 23 of *COL9A3*, which leads to a premature stop codon, has been reported in humans with STL type VI. These patients present with intellectual disability, hearing loss and mid face hypoplasia, and the only ocular phenotype reported is myopia [14]. Myopia is an ophthalmic feature not commonly tested in animals and was not assessed by retinoscopy in the present study. Interestingly, the phenotype of OSD-affected NID in the present study is actually more similar to other human ophthalmoarthropathies, including Kniest dysplasia (caused by mutations in *COL2A1****)*** [21, 22], Marshal syndrome (caused by mutations in *COL11A1****)*** [15, 23, 26] and finally STL types I, II, IV and V (caused by mutations in *COL2A1*, *COLL11A1*, *COL9A1* and *COL9A2* respectively) [12, 13, 15–20]. The severity of vitreal degeneration varied between OSD-affected NID which could have resembled the two types of congenital vitreous described in human STL types I and II. However, no deleterious variants were found in any of these genes in OSD-affected NID in this study.

In the present study, *COL9A3*_c.700C>T_ demonstrated an autosomal recessive mode of inheritance with complete penetrance i.e. all OSD-affected NID were homozygous for the variant, and all homozygotes were clinically affected. The mutant allele frequency observed in this study suggests that 15% of NID are carriers and <1% are clinically affected with the disease. However, these estimates are based on samples from the UK submitted to the AHT specifically for this research study and are unlikely to be representative of all NID. *COL9A3*_c.700C>T_ homozygotes will be clinically affected and can be recognised by a veterinarian or even an experienced breeder. However, *COL9A3*_c.700C>T_ heterozygotes can only be detected by genetic testing, and a DNA test has been developed and is available to the public, thereby providing breeders with a tool to prevent producing OSD-affected puppies. Pedigree analysis and tracing of the *COL9A3* mutant allele through NID pedigrees suggested that the variant either arose very early in the breed’s history or was inherited from one of the founding breeds. In addition, the NID breed is in turn one of the founding breeds of a number of other “wolf-like” breeds including the Utonagan, Tamaskan, British Timber dog and British Lupine dog. It is therefore possible that the *COL9A3*_c.700C>T_ variant could be present in any of these breeds. While it was not detected during this study, only small numbers of samples were tested and the possibility that the variant is present in one or more of these breeds cannot be excluded, particularly in the wolf-like breeds with NID ancestry. Indeed, through commercial DNA testing the AHT has tested a very small number of non-NID wolf-like dogs and identified a single heterozygous Utonagan. It is therefore suggested that it would be prudent of breeders of all wolf-like breeds to test for *COL9A3*_c.700C>T_.

## Conclusion

In the present study clinical, histopathological and genetic characterisation of OSD in NID is reported. All seven NID presented with short-limbed dwarfism, angular limb deformities and a combination of macroglobus, incipient to hypermature cataracts, lens coloboma, microphakia and vitreopathy, and a variety of other ocular signs were observed in some of the affected dogs. Using WGS and a candidate gene approach, a nonsense variant, *COL9A3*_c.700C>T_ (p.Arg234Ter), was identified which is strongly associated with autosomal recessive OSD in NID. The variant is predicted to result in a premature termination codon and a truncated protein. This study establishes a new canine OSD model, which could enable future investigations into the disease pathogenesis and development of potential therapies.

## Supporting information

S1 Table Primer and probe sequences. (XLSX)

S1 Appendix Reaction mixes and thermal cycling parameters. (DOCX)

S2 Appendix Quantitative PCR and Western blot. (DOCX)

## References

[1] Carrig CB, MacMillan A, Brundage S, Pool RR, Morgan JP. Retinal dysplasia associated with skeletal abnormalities in Labrador Retrievers. J Am Vet Med Assoc. 1977;170(1):49–57. Epub 1977/01/01. . 830631

[2] Carrig CB, Sponenberg DP, Schmidt GM, Tvedten HW. Inheritance of associated ocular and skeletal dysplasia in Labrador retrievers. J Am Vet Med Assoc. 1988;193(10):1269–72. Epub 1988/11/15. . 3204050

[3] Meyers VN, Jezyk PF, Aguirre GD, Patterson DF. Short-limbed dwarfism and ocular defects in the Samoyed dog. J Am Vet Med Assoc. 1983;183(9):975–9. Epub 1983/11/01. . 12002589

[4] Blair NP, Dodge JT, Schmidt GM. Rhegmatogenous retinal detachment in Labrador retrievers. I. Development of retinal tears and detachment. Arch Ophthalmol. 1985;103(6):842–7. 10.1001/archopht.1985.01050060102037 . 4004627

[5] Goldstein O, Guyon R, Kukekova A, Kuznetsova TN, Pearce-Kelling SE, Johnson J, et al COL9A2 and COL9A3 mutations in canine autosomal recessive oculoskeletal dysplasia. Mamm Genome. 2010;21(7–8):398–408. 10.1007/s00335-010-9276-4 20686772 PMC2954766

[6] Gelatt KN, Gilger B.C., Kern T.J. Veterinary ophthalmology / editor, Kirk N. Gelatt; associate editors, Brian C. Gilger, Thomas J. Kern. 5th ed ed. United States: Ames, Iowa: Wiley-Blackwell, 2013.; 2013.

[7] Aroch I, Ofri R, Aizenberg I. Haematological, ocular and skeletal abnormalities in a samoyed family. J Small Anim Pract. 1996;37(7):333–9. . 8840254 10.1111/j.1748-5827.1996.tb02403.x

[8] Blair NP, Dodge JT, Schmidt GM. Rhegmatogenous retinal detachment in Labrador retrievers. II. Proliferative vitreoretinopathy. Arch Ophthalmol. 1985;103(6):848–54. 10.1001/archopht.1985.01050060108038 . 4004628

[9] Ahmad NN, Ala-Kokko L, Knowlton RG, Jimenez SA, Weaver EJ, Maguire JI, et al Stop codon in the procollagen II gene (COL2A1) in a family with the Stickler syndrome (arthro-ophthalmopathy). Proc Natl Acad Sci U S A. 1991;88(15):6624–7. 10.1073/pnas.88.15.6624 1677770 PMC52140

[10] Richards AJ, Yates JR, Williams R, Payne SJ, Pope FM, Scott JD, et al A family with Stickler syndrome type 2 has a mutation in the COL11A1 gene resulting in the substitution of glycine 97 by valine in alpha 1 (XI) collagen. Hum Mol Genet. 1996;5(9):1339–43. 10.1093/hmg/5.9.1339 . 8872475

[11] Sirko-Osadsa DA, Murray MA, Scott JA, Lavery MA, Warman ML, Robin NH. Stickler syndrome without eye involvement is caused by mutations in COL11A2, the gene encoding the alpha2(XI) chain of type XI collagen. J Pediatr. 1998;132(2):368–71. 10.1016/s0022-3476(98)70466-4 . 9506662

[12] Nikopoulos K, Schrauwen I, Simon M, Collin RW, Veckeneer M, Keymolen K, et al Autosomal recessive Stickler syndrome in two families is caused by mutations in the COL9A1 gene. Invest Ophthalmol Vis Sci. 2011;52(7):4774–9. 10.1167/iovs.10-7128 . 21421862

[13] Baker S, Booth C, Fillman C, Shapiro M, Blair MP, Hyland JC, et al A loss of function mutation in the COL9A2 gene causes autosomal recessive Stickler syndrome. Am J Med Genet A. 2011;155A(7):1668–72. 10.1002/ajmg.a.34071 . 21671392

[14] Faletra F, D'Adamo AP, Bruno I, Athanasakis E, Biskup S, Esposito L, et al Autosomal recessive Stickler syndrome due to a loss of function mutation in the COL9A3 gene. Am J Med Genet A. 2014;164A(1):42–7. 10.1002/ajmg.a.36165 . 24273071

[15] Annunen S, Korkko J, Czarny M, Warman ML, Brunner HG, Kaariainen H, et al Splicing mutations of 54-bp exons in the COL11A1 gene cause Marshall syndrome, but other mutations cause overlapping Marshall/Stickler phenotypes. Am J Hum Genet. 1999;65(4):974–83. 10.1086/302585 10486316 PMC1288268

[16] Martin S, Richards AJ, Yates JR, Scott JD, Pope M, Snead MP. Stickler syndrome: further mutations in COL11A1 and evidence for additional locus heterogeneity. Eur J Hum Genet. 1999;7(7):807–14. 10.1038/sj.ejhg.5200377 . 10573014

[17] Van Camp G, Snoeckx RL, Hilgert N, van den Ende J, Fukuoka H, Wagatsuma M, et al A new autosomal recessive form of Stickler syndrome is caused by a mutation in the COL9A1 gene. Am J Hum Genet. 2006;79(3):449–57. 10.1086/506478 16909383 PMC1559536

[18] Robin NH, Moran RT, Ala-Kokko L. Stickler Syndrome In: Adam MP, Ardinger HH, Pagon RA, Wallace SE, Bean LJH, Stephens K, et al, editors. GeneReviews((R)) Seattle (WA) 2017.

[19] Vu CD, Brown J Jr., Korkko J, Ritter R 3rd, Edwards AO. Posterior chorioretinal atrophy and vitreous phenotype in a family with Stickler syndrome from a mutation in the COL2A1 gene. Ophthalmology. 2003;110(1):70–7. 10.1016/s0161-6420(02)01446-x . 12511349

[20] Stickler GB, Belau PG, Farrel FJ, Jones JD, Pugh DG, Steinberg AG, et al Hereditary Progressive Arthro-Ophthalmology. Mayo Clin Proc. 1965;40:433–55. . 14299791

[21] Kniest W. [Differential diagnosis between dysostosis enchondralis and chondrodystrophy]. Z Kinderheilkd. 1952;70(6):633–40. . 12995812

[22] Maumenee IH, Traboulsi EI. The ocular findings in Kniest dysplasia. Am J Ophthalmol. 1985;100(1):155–60. 10.1016/s0002-9394(14)74998-0 . 4014370

[23] Griffith AJ, Sprunger LK, Sirko-Osadsa DA, Tiller GE, Meisler MH, Warman ML. Marshall syndrome associated with a splicing defect at the COL11A1 locus. Am J Hum Genet. 1998;62(4):816–23. 10.1086/301789 9529347 PMC1377029

[24] Majava M, Hoornaert KP, Bartholdi D, Bouma MC, Bouman K, Carrera M, et al A report on 10 new patients with heterozygous mutations in the COL11A1 gene and a review of genotype-phenotype correlations in type XI collagenopathies. Am J Med Genet A. 2007;143A(3):258–64. 10.1002/ajmg.a.31586 . 17236192

[25] Ayme S, Preus M. The Marshall and Stickler syndromes: objective rejection of lumping. J Med Genet. 1984;21(1):34–8. 10.1136/jmg.21.1.34 6694183 PMC1049203

[26] Marshall D. Ectodermal dysplasia; report of kindred with ocular abnormalities and hearing defect. Am J Ophthalmol. 1958;45(4 Pt 2):143–56. . 13520885

[27] Simon L. Northern Inuit Dog: Dog Zone. Available from: http://www.dogzone.com/breeds/northern-inuit-dog/.

[28] Rozen S, Skaletsky H. Primer3 on the WWW for general users and for biologist programmers. Methods Mol Biol. 2000;132:365–86. 10.1385/1-59259-192-2:365 . 10547847

[29] Li H, Durbin R. Fast and accurate short read alignment with Burrows-Wheeler transform. Bioinformatics. 2009;25(14):1754–60. 10.1093/bioinformatics/btp324 19451168 PMC2705234

[30] McKenna A, Hanna M, Banks E, Sivachenko A, Cibulskis K, Kernytsky A, et al The Genome Analysis Toolkit: a MapReduce framework for analyzing next-generation DNA sequencing data. Genome Res. 2010;20(9):1297–303. 10.1101/gr.107524.110 20644199 PMC2928508

[31] Mayne R, Ren ZX, Liu J, Cook T, Carson M, Narayana S. VIT-1: the second member of a new branch of the von Willebrand factor A domain superfamily. Biochem Soc Trans. 1999;27(6):832–5. 10.1042/bst0270832 . 10830112

[32] Reardon AJ, Le Goff M, Briggs MD, McLeod D, Sheehan JK, Thornton DJ, et al Identification in vitreous and molecular cloning of opticin, a novel member of the family of leucine-rich repeat proteins of the extracellular matrix. J Biol Chem. 2000;275(3):2123–9. 10.1074/jbc.275.3.2123 . 10636917

[33] Hobby P, Wyatt MK, Gan W, Bernstein S, Tomarev S, Slingsby C, et al Cloning, modeling, and chromosomal localization for a small leucine-rich repeat proteoglycan (SLRP) family member expressed in human eye. Mol Vis. 2000;6:72–8. . 10837509

[34] Friedman JS, Ducharme R, Raymond V, Walter MA. Isolation of a novel iris-specific and leucine-rich repeat protein (oculoglycan) using differential selection. Invest Ophthalmol Vis Sci. 2000;41(8):2059–66. . 10892843

[35] Frischknecht M, Niehof-Oellers H, Jagannathan V, Owczarek-Lipska M, Drogemuller C, Dietschi E, et al A COL11A2 mutation in Labrador retrievers with mild disproportionate dwarfism. PLoS One. 2013;8(3):e60149 10.1371/journal.pone.0060149 23527306 PMC3603880

[36] Mayne R, Brewton RG, Mayne PM, Baker JR. Isolation and characterization of the chains of type V/type XI collagen present in bovine vitreous. J Biol Chem. 1993;268(13):9381–6. . 8486632

[37] van Steensel MA, Buma P, de Waal Malefijt MC, van den Hoogen FH, Brunner HG. Oto- spondylo-megaepiphyseal dysplasia (OSMED): clinical description of three patients homozygous for a missense mutation in the COL11A2 gene. Am J Med Genet. 1997;70(3):315–23. . 9188673 10.1002/(sici)1096-8628(19970613)70:3<315::aid-ajmg19>3.3.co;2-y

[38] Pellegrini B, Acland GM, Ray J. Cloning and characterization of opticin cDNA: evaluation as a candidate for canine oculo-skeletal dysplasia. Gene. 2002;282(1–2):121–31. 10.1016/s0378-1119(01)00842-3 . 11814684

[39] Sim NL, Kumar P, Hu J, Henikoff S, Schneider G, Ng PC. SIFT web server: predicting effects of amino acid substitutions on proteins. Nucleic Acids Res. 2012;40(Web Server issue):W452–7. 10.1093/nar/gks539 22689647 PMC3394338

[40] Adzhubei I, Jordan DM, Sunyaev SR. Predicting functional effect of human missense mutations using PolyPhen-2. Curr Protoc Hum Genet. 2013;Chapter 7:Unit7 20. 10.1002/0471142905.hg0720s76 23315928 PMC4480630

[41] Reese MG, Eeckman FH, Kulp D, Haussler D. Improved splice site detection in Genie. J Comput Biol. 1997;4(3):311–23. 10.1089/cmb.1997.4.311 . 9278062

[42] Bonfield JK, Smith K, Staden R. A new DNA sequence assembly program. Nucleic Acids Res. 1995;23(24):4992–9. 10.1093/nar/23.24.4992 8559656 PMC307504

[43] Livak KJ, Schmittgen TD. Analysis of relative gene expression data using real-time quantitative PCR and the 2(-Delta Delta C(T)) Method. Methods. 2001;25(4):402–8. 10.1006/meth.2001.1262 . 11846609

[44] Chiwitt CLH, Baines SJ, Mahoney P, Tanner A, Heinrich CL, Rhodes M, et al Ocular biometry by computed tomography in different dog breeds. Vet Ophthalmol. 2017;20(5):411–9. 10.1111/vop.12441 . 27862797

[45] Robinson JT, Thorvaldsdottir H, Winckler W, Guttman M, Lander ES, Getz G, et al Integrative genomics viewer. Nat Biotechnol. 2011;29(1):24–6. Epub 2011/01/12. 10.1038/nbt.1754 21221095 PMC3346182

[46] Gathwala G, Bhalla K, Dalal JS, Arya V. Stickler Syndrome with bilateral corneal opacities—A rare entity. Int J Bas Appl Med Sci. 2013;3(1 (January-March)):8–9.

[47] Goyal M, Kapoor S, Ikegawa S, Nishimura G. Stickler Syndrome Type 1 with Short Stature and Atypical Ocular Manifestations. Case Rep Pediatr. 2016;2016:3198597 10.1155/2016/3198597 28018693 PMC5149639

[48] Schepens CL. Clinical aspects of pathologic changes in the vitreous body. Am J Ophthalmol. 1954;38(1:2):8–21. 10.1016/0002-9394(54)90004-5 . 13180614

[49] Teng CC, Chi HH. Vitreous changes and the mechanism of retinal detachment. Am J Ophthalmol. 1957;44(3):335–56. 10.1016/0002-9394(57)92766-6 . 13458300

[50] Edwards AO. Clinical features of the congenital vitreoretinopathies. Eye (Lond). 2008;22(10):1233–42. 10.1038/eye.2008.38 . 18309337

[51] Plummer CE, Regnier A, Gelatt KN. The Canine Glaucomas In: Gelatt KN, Gilger BC, Kern TJ, editors. Veterinary Ophthalmology. 174. 5th ed: John Wiley & Sons; 2013 p. 1050–128.

[52] Gelse K, Poschl E, Aigner T. Collagens—structure, function, and biosynthesis. Adv Drug Deliv Rev. 2003;55(12):1531–46. . 14623400 10.1016/j.addr.2003.08.002

[53] Mendler M, Eich-Bender SG, Vaughan L, Winterhalter KH, Bruckner P. Cartilage contains mixed fibrils of collagen types II, IX, and XI. J Cell Biol. 1989;108(1):191–7. 10.1083/jcb.108.1.191 2463256 PMC2115367

[54] van der Rest M, Mayne R, Ninomiya Y, Seidah NG, Chretien M, Olsen BR. The structure of type IX collagen. J Biol Chem. 1985;260(1):220–5. . 2981204

[55] van der Rest M, Mayne R. Type IX collagen proteoglycan from cartilage is covalently cross-linked to type II collagen. J Biol Chem. 1988;263(4):1615–8. . 3123475

[56] Shaw LM, Olsen BR. FACIT collagens: diverse molecular bridges in extracellular matrices. Trends Biochem Sci. 1991;16(5):191–4. . 1882421 10.1016/0968-0004(91)90074-6

[57] Bishop MA, Steiner JM, Moore LE, Williams DA. Evaluation of the cationic trypsinogen gene for potential mutations in miniature schnauzers with pancreatitis. Can J Vet Res. 2004;68(4):315–8. 15581228 PMC1111364

[58] Olsen BR. Collagen IX. Int J Biochem Cell Biol. 1997;29(4):555–8. . 9363632 10.1016/s1357-2725(96)00100-8

[59] Wu JJ, Woods PE, Eyre DR. Identification of cross-linking sites in bovine cartilage type IX collagen reveals an antiparallel type II-type IX molecular relationship and type IX to type IX bonding. J Biol Chem. 1992;267(32):23007–14. . 1429648

[60] Bishop PN, Holmes DF, Kadler KE, McLeod D, Bos KJ. Age-related changes on the surface of vitreous collagen fibrils. Invest Ophthalmol Vis Sci. 2004;45(4):1041–6. 10.1167/iovs.03-1017 . 15037566

[61] Yu Y, Fuscoe JC, Zhao C, Guo C, Jia M, Qing T, et al A rat RNA-Seq transcriptomic BodyMap across 11 organs and 4 developmental stages. Nat Commun. 2014;5:3230 10.1038/ncomms4230 24510058 PMC3926002

[62] Yue F, Cheng Y, Breschi A, Vierstra J, Wu W, Ryba T, et al A comparative encyclopedia of DNA elements in the mouse genome. Nature. 2014;515(7527):355–64. 10.1038/nature13992 25409824 PMC4266106

