# Supplementary material for: Clinical, histopathological and genetic characterisation of oculoskeletal dysplasia in the Northern Inuit Dog
Source: PLoS One. 2019 Aug 15;14(8):e0220761. doi: 10.1371/journal.pone.0220761 (PMC6695176; doi:10.1371/journal.pone.0220761)
Supplement: S1 Appendix — (DOCX) [file pone.0220761.s002.docx]

**S1 Appendix. Reaction mixes and thermal cycling parameters**

**Screening of *drd1* and *drd2* mutations**

***Drd1*** (dwarfism with retinal dysplasia 1)

Reactions were carried out in 12 μL volumes consisting of 0.2 mM dNTPs (ThermoFisher Scientific), 1x PCR buffer, 1x Q solution and 0.05 U/uL HotStarTaq Plus DNA Polymerase (Qiagen), 0.83 μM each primer (S1 Table) and 2 μL genomic DNA. Cycling parameters were 98°C for 5 min, 35 cycles of 98°C for 30 sec, 58°C for 30 sec and 72°C for 90 sec, and finally 72°C for 5 min. PCR amplification was carried out on an SimpliAmp (Applied Biosystems) or T100 (BioRad) thermal cycler and amplicon sizes compared by agarose gel electrophoresis using a 2-log DNA ladder (New England Biolabs).

***Drd2* (**dwarfism with retinal dysplasia 2)

Reactions were carried out in 12 μL volumes consisting of 0.2 mM dNTPs (ThermoFisher Scientific), 1x PCR buffer and 0.1 U/uL HotStarTaq Plus DNA Polymerase (Qiagen), 0.17 μM forward primer, 0.42 uM reverse primer, 0.5 uM fluorescent primer (S1 Table) and 2 μL genomic DNA. Cycling parameters were 94°C for 4 min, 30 cycles of 94°C for 1 min, 66°C for 1 min and 72°C for 1 min, 8 cycles of 94°C for 1 min, 50°C for 1 min and 72°C for 1 min, and finally 72°C for 30 min. PCR amplification was carried out on an SimpliAmp (Applied Biosystems) or T100 (BioRad) thermal cycler and amplicon sizes compared by capillary electrophoresis a 3130xl Genetic Analyzer (Applied Biosystems).

**Northern Inuit Dog Oculoskeletal dysplasia variant Sanger sequencing**

PCR amplification: Reactions were carried out in 12 μL volumes consisting of 0.2 mM dNTPs (ThermoFisher Scientific), 1x PCR buffer and 0.1 U/uL HotStarTaq Plus DNA Polymerase (Qiagen), 0.83 μM each primer (S1 Table) and 2 μL genomic DNA. Cycling parameters were 95°C for 5 min, 35 cycles of 95°C for 30 sec, 57°C for 30 sec and 72°C for 30 sec, and finally 72°C for 5 min. PCR amplification was carried out on an SimpliAmp (Applied Biosystems) or T100 (BioRad) thermal cycler. PCR products were purified using a MultiScreen-PCR96 Filter Plate (Merck). Sanger sequencing reactions were carried out in 6 μL volumes consisting of 0.5 uL BigDye v3.1 (Applied Biosystems), 1 uL sequencing buffer, 1 uL primer (2 uM), 1.5 uL ultrapure water and 2 uL PCR product. Cycling parameters were 96°C for 3 min, 44 cycles of 92°C for 4 sec, 55°C for 4 sec and 60°C for 4 sec. Thermal cycling was carried out on an SimpliAmp (Applied Biosystems) or T100 (BioRad) thermal cycler and sequencing products were purified using a MultiScreen-PCRµ96 Filter Plate (Merck). Amplicons were sequenced on a 3130xl Genetic Analyser.
