# Supplementary material for: Clinical, histopathological and genetic characterisation of oculoskeletal dysplasia in the Northern Inuit Dog
Source: PLoS One. 2019 Aug 15;14(8):e0220761. doi: 10.1371/journal.pone.0220761 (PMC6695176; doi:10.1371/journal.pone.0220761)
Supplement: S2 Appendix — (DOCX) [file pone.0220761.s003.docx]

### S2 Appendix. Quantitative PCR and Western Blot

### Agarose gel electrophoresis of qPCR products


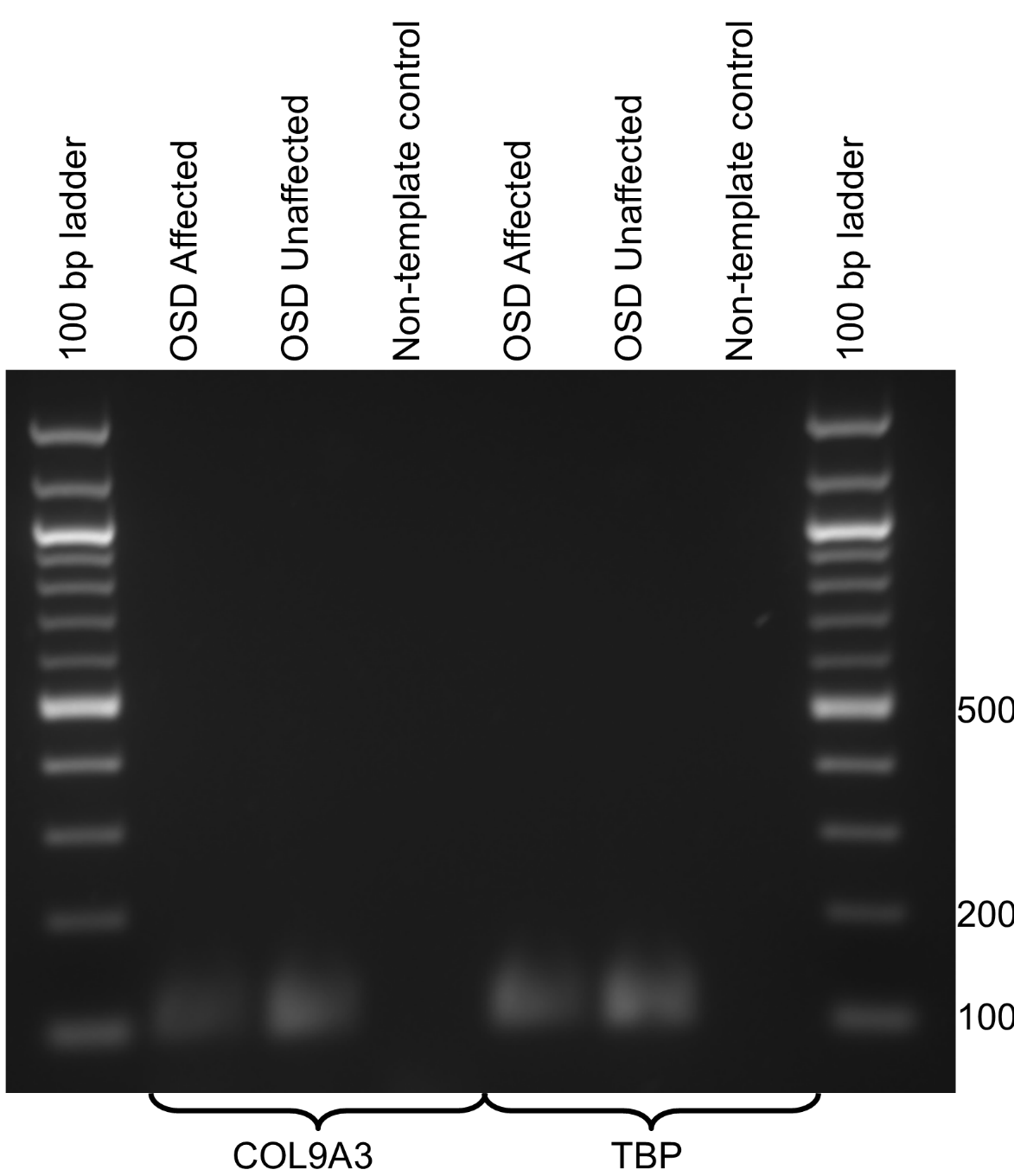


### Agarose gel electrophoresis of the final qPCR products. Products generated from *COL9A3* and *TBP* primers were both around 100 bp, as expected from the amplification of mRNA. Amplification of gDNA would result in product sizes of approximately 650bp and 6200bp respectively.

### Western blot analysis

**Methods**

Protein was extracted from retinal tissue using RIPA buffer, and concentrations determined using the Qubit^TM^ Protein Assay Kit (ThermoFisher Scientific). A total of 80 µg protein, along with 10 uL Pre-stained protein ladder (New England Biolabs) and 10 uL HeLa whole cell lysate (Abcam, ab150035), was separated on a 4–20% Mini-PROTEAN® TGX Stain-Free™ Protein Gels (BioRad) under reducing conditions, and immunoblotted on a 0.45µm nitrocellulose membrane (BioRad). Protein-blotted membranes were probed with anti-*COL9A3* (1:1000; Abnova, H00001299-D01P; raised against full-length human *COL9A3* protein) and anti-GAPDH (1:2500; Abcam, ab9485) rabbit polyclonal antibodies. *COL9A3* and GAPDH proteins were detected using the WesternBreeze^TM^ anti-rabbit Chromogenic Kit according to the manufacturer’s instructions.

**Results**

***COL9A3* protein is not detected in OSD-affected NID**

Western blotting was carried out on retinal protein from an OSD-affected NID (Dog 2, 18 months old), an OSD-unaffected Golder Retriever with healthy eyes (10 years old) and an OSD-unaffected Irish Setter with epilepsy but unknown ocular clinical status (four years old), using antibodies against *COL9A3* and GAPDH. Interestingly, a 50 kDa band consistent with *COL9A3* was observed in only the Irish Setter control, but not in either the Golden Retriever control or the OSD-affected NID. A smaller 6 kDa band consistent with mutant *COL9A3* was also not observed.


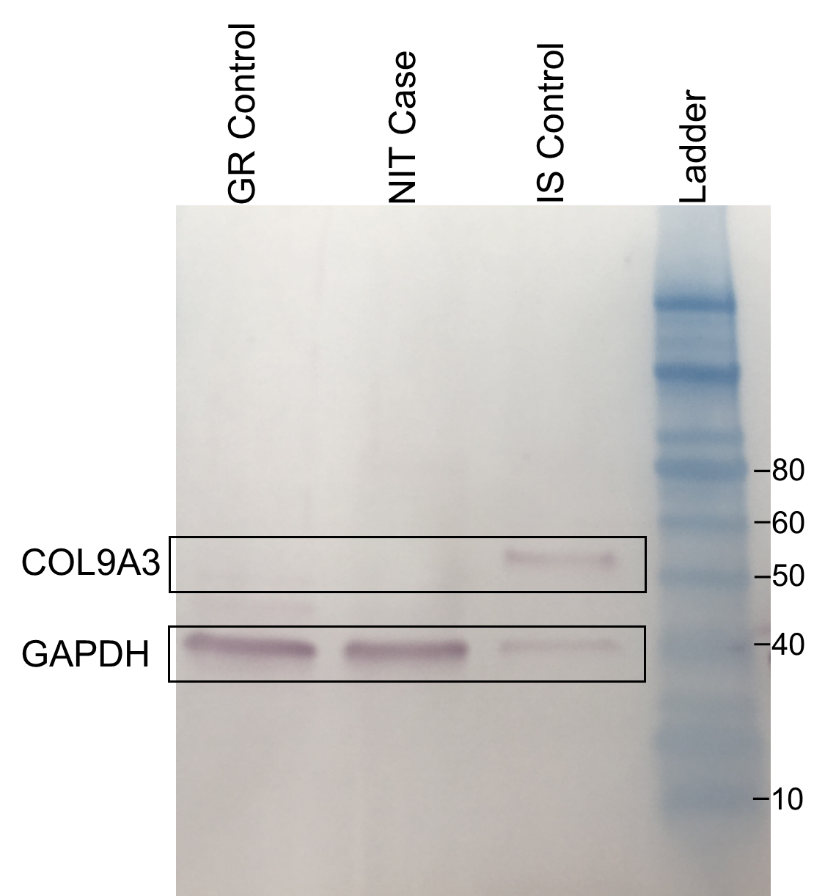


**Western blot:** GAPDH loading control, expected to be 37 kDa, is observed in all three samples. *COL9A3* protein, expected to be 50 or 75 kDa, is observed in the younger (four year old) Irish Setter control, but not in the older (10 year old) Golden Retriever control or in the OSD-affected NID (case). The smaller (truncated) *COL9A3* protein, expected to be 6 kDa, is also not observed in the OSD-affected NID. Ladder = Pre-stained protein ladder (New England Biolabs).

**Discussion**

As discussed in the manuscript, mouse and rat studies have reported maximum *COL9A3* expression in juvenile animals, with much less in adolescent and even less in adult animals. Our observation that *COL9A3* protein was detected by western blot in a four year old OSD-unaffected dog, but not in a 10 year old OSD-unaffected dog supports this hypothesis. Mutant *COL9A3*, if expressed, is expected to be around 6 kDa in size, but was also not detected by western blot in the 18 month old OSD-affected NID. It is possible that the protein is in fact expressed but is not detected by the antibody used due to changes in structure or loss of antibody-specific epitopes. Although the antibody was raised against the full-length human COL9A3 protein, it is possible that the dominant epitopes are latter part of the protein that is expected to be lost, and are therefore not present to be detected by the antibody. This provides a hypothesis to explain why NID heterozygotes did not show an ocular phenotype similar to that seen in some skeletally normal Labrador Retriever heterozygotes. In Labrador Retrievers the mutant *COL9A3* mRNA undergoes nonsense-mediated decay and as a result no protein is produced. Heterozygous dogs therefore will have only around 50% functional *COL9A3* protein, which could be the approximate minimum required for optimal and healthy ocular function. On the other hand, OSD-affected NID could still have some protein produced from a mutant allele which functions sufficiently enough for normal ocular needs when supplemented by normal protein. Nevertheless, the observation that *COL9A3* protein expression in the OSD-affected NID differs drastically from that of the four year old OSD-unaffected non-NID supports the hypothesis that *COL9A3*c.700 >T is the OSD-causing variant in NID.
